# Crystallographic and EPR-based characterisation of Cu^2+^-binding to serum albumin: ATCUN coordination and additional sites

**DOI:** 10.1039/d6qi00150e

**Published:** 2026-03-12

**Authors:** Michal Gucwa, Katarzyna B. Handing, Vanessa Bijak, Katrin Ackermann, Aisika Chakraborty, Anastasiya Pautarak, Timothy Redpath, Boyang Lin, Joanna Slawek, Claudia A. Blindauer, Alan J. Stewart, Bela E. Bode, Wladek Minor

**Affiliations:** a Department of Molecular Physiology and Biological Physics, University of Virginia School of Medicine Charlottesville VA 22908-0736 USA wladek@minorlab.org; b SOLARIS, National Synchotron Radiation Centre, Jagiellonian University Czerwone Maki 98 30-392 Kraków Poland; c Department of Computational Biophysics and Bioinformatics, Jagiellonian University Kraków Poland; d EaStCHEM School of Chemistry, Biomedical Sciences Research Complex, and Centre of Magnetic Resonance, University of St Andrews St Andrews KY16 9ST UK beb2@st-andrews.ac.uk; e School of Medicine, Biomedical Sciences Research Complex, and Centre of Magnetic Resonance, University of St Andrews St Andrews KY16 9TF UK ajs21@st-andrews.ac.uk; f Department of Chemistry, University of Warwick Coventry CV4 7SH UK

## Abstract

Copper homeostasis is essential for mammalian physiology. Serum albumin plays an important role in plasma copper transport and buffering, yet its Cu^2+^ binding sites have remained incompletely characterised. Here we report the first X-ray crystal structure of a mammalian serum albumin, in this case equine albumin, bound to copper(ii). The structure revealed a high-affinity ATCUN site with characteristic square-planar geometry. Additional Cu^2+^ binding was observed at five secondary sites, including sites A and B and other histidine-containing sites (involving either His287, His317 and His509). Continuous-wave EPR spectroscopy further supported a square-planar coordination at the ATCUN site through a low-*g* spectral feature appearing upon binding of less than one molar equivalent of Cu^2+^. ESEEM and HYSCORE experiments detected nuclear quadrupole interactions and weakly coupled ^14^N signals, supporting histidine involvement and increased water coordination at higher Cu^2+^ loading. RIDME-derived distance distributions and structural simulations indicate simultaneous occupancy of multiple sites, with strong evidence for ATCUN and site B, and partial engagement of distal histidines (His287, His317) at elevated Cu^2+^ equivalents. These findings support a dynamic, multi-site binding model in which short-range distances arise from ATCUN and site B co-occupancy, while longer-range peaks reflect contributions from distal histidine sites.

## Abstract 2

This work presents the first X-ray crystal structure of a mammalian serum albumin bound to copper(ii), revealing dynamic, multi-site binding that integrates a square-planar ATCUN site with secondary histidine-containing sites.

## Graphical abstract

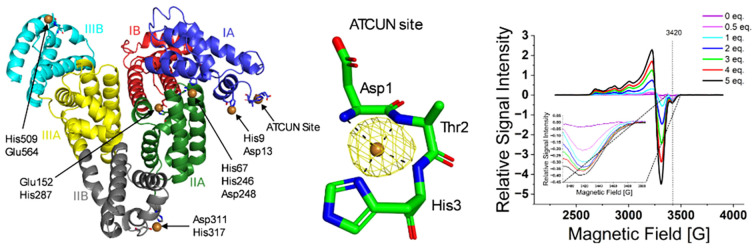

## 1. Introduction

Serum albumin is the most abundant protein in plasma and plays a key role in transporting various endogenous and exogenous substances, including metal ions.^1^ Although ceruloplasmin is the principal Cu^2+^-binding protein in plasma,^2^ the interaction between Cu^2+^ and albumin is of high importance and helps to regulate free Cu^2+^ levels, preventing oxidative damage due to copper's redox activity and facilitating cell uptake *via* human copper transporter 1 (hCtr1).^3,4^

Albumin binds Cu^2+^ primarily at the amino-terminal copper/nickel site (ATCUN motif) with high affinity and specificity (*K* = 10^16^).^5^ Ni^2+^ also binds at this site, though with significantly lower affinity (*K* = 3.98 × 10^9^).^6^ The ATCUN site was first implicated in Cu^2+^ binding in 1960, when copper(ii) chloride was found to block the albumin amino group from reacting with Sanger's reagent, fluorodinitrobenzene.^7^ Subsequent peptide studies demonstrated that Cu^2+^ binds strongly to fragments corresponding to residues 1–24 of bovine serum albumin (BSA) and to the tetrapeptide Asp–Thr–His–Lys (residues 1–4), with affinities comparable to intact BSA.^8^ Later, ^1^H NMR analyses confirmed the presence of a Cu^2+^/Ni^2+^ site and suggested a square-planar coordination geometry.^9^

Secondary Cu^2+^-binding sites on albumin also exist, which are likely to contribute to Cu^2+^ binding and clearance upon overload. Recently, pulsed electron paramagnetic resonance (EPR) spectroscopy experiments have suggested that secondary Cu^2+^ sites on human serum albumin (HSA) involve His9 and His288 residues.^10^ Over the last decade or so, several metal ion binding sites on albumin have been structurally characterised, including those for Zn^2+^,^11^ Co^2+^,^12^ and Ca^2+^.^13^ In addition to free metal ions, several studies have reported complexes formed with metal-containing compounds, most notably thiosemicarbazone-derived complexes, including those of Cu(ii).^14^ However, to date, structures capturing the binding of free Cu^2+^ ions to serum albumin at its physiological metal-binding sites have not been elucidated. A summary of albumin structures containing metal ions and metal containing compounds can be found in Table S1.

In this study, we report the X-ray crystal structure of equine serum albumin (ESA) in complex with Cu^2+^, providing detailed insights into the locations and coordination environments of its copper-binding sites. ESA was selected for these experiments as it crystallises more readily than HSA and yields higher-resolution diffraction data in the absence of stabilising fatty acids (Fig. S1). Moreover, the Cu^2+^-coordinating residues are highly conserved between ESA and HSA, supporting the suitability of ESA as a structural model. To complement these structural findings, we employed pulsed EPR spectroscopy, enabling a more detailed examination of the individual contributions of the Cu^2+^ sites within ESA.

## 2. Experimental

### 2.1. Materials

For X-ray crystallography, ESA isolated from natural sources was purchased from Equitech-Bio (Kerrville, TX, USA; #ESA62) as lyophilised powder and purified further as described below. For EPR experiments, ESA was solvent extracted from horse plasma. The final protein purity was above 95% as assessed by SDS-PAGE and gel filtration chromatography.

### 2.2. ESA purification and crystallisation

ESA was dissolved in purification buffer (10 mM Tris-HCl, 150 mM NaCl, pH 7.5) and purified by size-exclusion chromatography on a Superdex 200 column using the same buffer as the mobile phase. All purification steps were performed at 4 °C using an ÄKTA FPLC system (Cytiva). Fractions corresponding to the monomeric species (∼55–60 kDa) were pooled and concentrated with an Amicon Ultra centrifugal filter (30 kDa MWCO). The protein concentration was determined by measuring absorbance at 280 nm with a Shimadzu UV-2450 spectrophotometer (Kyoto, Japan). The concentration was then adjusted to reach a final concentration of approximately 50 mg mL^−1^. The homogeneity and integrity of the purified protein were verified by SDS-polyacrylamide gel electrophoresis.

Crystals of ESA in complex with Cu^2+^ were obtained using the sitting-drop vapor diffusion method in 96-well plates. Plates were set up using a Mosquito crystallisation robot (TTP Labtech, Cambridge, MA) by mixing 200 nL of ESA protein solution (36 mg mL^−1^) with 200 nL of reservoir solution in a 1 : 1 ratio. The reservoir solution contained 0.2 M Li_2_SO_4_, 0.1 M Tris-HCl (pH 7.4), 2.2 M (NH_4_)_2_SO_4_, and 5 mM CuCl_2_, yielding a final Cu^2+^ concentration of approximately 2.5 mM in the crystallisation drop. The crystallisation plates were incubated at room temperature, and Paratone-N oil was used as the cryoprotectant.

### 2.3. Data collection and structure determination

X-ray diffraction data were collected from single crystals of the Cu^2+^–ESA complex at 100 K at the SBC-CAT 19-BM beamline at the Advanced Photon Source, Argonne National Laboratory (Argonne, IL). Data were collected at the selenium absorption K-edge (12 670 eV), where anomalous diffraction from Cu^2+^ ions remained detectable. Diffraction images were processed and scaled using HKL-3000,^15,16^ in scale anomalous mode to enable calculation of anomalous difference maps for Cu^2+^. The structures were solved by molecular replacement with the ESA structure (Protein Data Bank (PDB) ID: 7mbl)^12^ as the search model. Initial electron density maps and preliminary models were generated in HKL-3000, which integrates MOLREP,^17^ and other programs from the CCP4 suite.^18^ Iterative cycles of model building and refinement were performed in COOT,^19^ and REFMAC,^20^ through the HKL-3000 interface. The final model quality was assessed using MolProbity,^21^ both as a standalone tool and within HKL-3000, as well as with wwPDB validation tools.^22^ The statistics for diffraction data collection, structure refinement, and structure quality are summarised in Table 1.

**Table 1:** Data collection, structure refinement, and structure quality statistics. Values in parentheses correspond to the highest resolution shell of the scaled dataset. Values in square brackets refer to the highest resolution shell included in refinement. Ramachandran plot statistics are calculated by MolProbity

PDB ID	9zmd
Diffraction images DOI	https://dx.doi.org/10.18430/M39ZMD
Data collection resolution (Å)	28.00–2.36
	(2.40–2.36)
	[2.66–2.60]
Refinement resolution (Å)	26.98–2.65
Wavelength (Å)	1.28291
Space group	*P*61
Unit-cell dimensions: *a*, *b*, *c* (Å)	93.43, 93.43, 141.75
Angles: *α*, *β*, *γ* (°)	90, 90, 120
Protein chains in the ASU	1
Completeness (%)	98.8 (90.4) [99.7]
Number of unique reflections	155 071
Redundancy	5.5 (3.5) [5.7]
〈*I*〉/〈*σ*(*I*)〉	17.7 (0.8)
CC ½	(0.48) [0.88]
*R*_merge_	0.081 (0.919) [0.641]
*R*_pim_	0.037 (0.518) [0.287]
*R*_work_/R_free_	0.191/0.251
Bond lengths RMSD (Å)	0.003
Bond angles RMSD (°)	1.07
Mean ADP (Å^2^)	38
Mean ADP for ligands/ions (Å^2^)	88
Number of protein atoms	4607
Mean ADP for protein (Å^2^)	38
Number of water molecules	230
Mean ADP for water molecules (Å^2^)	33
Clashscore	2.51
MolProbity score	1.34
Sidechain outliers (%)	1.97
Ramachandran outliers (%)	0.17
Ramachandran favored (%)	97.59

### 2.4. EPR sample preparation

Stock solutions of ∼1 mM ESA were prepared by resuspension of freeze-dried protein in protonated or deuterated buffer (50 mM Tris (Merck), 50 mM NaCl (Fisher Scientific), pH 7.4). The albumin concentration was calculated by measuring the absorbance at 280 nm. Stock solutions of CuCl_2_ (Thermo Scientific Chemicals) at 10 mM concentration were prepared in D_2_O (Merck) for the deuterated sample and H_2_O for protonated samples. Samples were prepared with a final volume of 65 µL, at 250 µM final protein concentration, and 50% cryoprotectant (protonated glycerol (Alfa Aesar) or deuterated glycerol (Cortecnet)). For the protonated samples, different concentrations of CuCl_2_ (0 to 5 molar equivalents) were added, the deuterated sample contained 2 molar equivalents of CuCl_2_. All samples were immediately snap-frozen in liquid nitrogen.

### 2.5. Continuous wave (CW) EPR

CW EPR spectra of the protonated samples were obtained at 120 K with a Bruker EMX plus spectrometer running Xenon software and equipped with an ELEXSYS Super Hi-Q resonator at an operating frequency of ∼9.5 GHz with 100 kHz modulation. The temperature was controlled by an ER4141 VT nitrogen variable temperature unit (Bruker). CW spectra of the pseudo-titration samples (0 to 5 molar equivalents of Cu^2+^) were recorded centred at 3100 G with a field sweep of 1600 G and a time constant of 20.48 ms, a conversion time of 20.62 ms, with 2667 points resolution. An attenuation of 10.0 dB (20 mW power) and modulation amplitude of 3 G were used for all spectra. The spectra were background and phase corrected. The double integral was obtained using the Xenon software. Simulations and fitting of CW EPR spectra were performed using pepper function in Easyspin.^23^

### 2.6. Pulse EPR

Pulse EPR experiments were performed at X-band (9 GHz) and at Q-band (34 GHz) frequencies on Bruker ELEXSYS E580 spectrometers, equipped with probe heads bearing a split-ring resonator (4118X-MS3) at X-band and a 3 mm cylindrical resonator (ER 5106QT-2w) at Q-band. Pulses were amplified by pulse travelling wave tube amplifiers (Applied Systems Engineering) with output of 150 W and 1 kW at Q- and X-band respectively. Temperature was controlled by cryogen free variable temperature cryostats (Cryogenic Ltd) operating at temperature range of 1.8–300 K.

### 2.7. Hyperfine spectroscopy (protonated samples)

The 3-Pulse electron spin echo envelope modulation (ESEEM)^24–26^ spectra were recorded at X-band at 30 K at the field positions corresponding to the maxima of the field swept spectra for two samples with 250 µM ESA and 0.5 and 5 molar equivalents of Cu^2+^, respectively. A pulse length of 16 ns for π/2 pulses with an inter pulse delay *τ* corresponding to a ^1^H blind spot (∼220 ns analogous to ∼3 times the inverse of the proton Larmor frequency, with *τ*_1(H)_ = 73 ns) was used. The delay time ‘T’ was set as 280 ns and incremented with a step of 8 ns and a 4-step phase cycle was employed. Four *τ* values were recorded, where the inter-pulse delay was incremented by 36 ns (0.5 × *τ*_1(H)_); the 3rd *τ* (292 ns) was selected for the HYSCORE experiment. The data were analysed by fitting an exponential background decay function to the phase corrected data and subtracting this background from the data before dividing the difference by the same background function, thus retaining information about the amplitude after fast Fourier transformation (FFT).^27^ The resulting trace was then subjected to a Hamming window, zero-filling and FFT, before the absolute (or magnitude) spectrum was obtained.

HYSCORE,^28^ spectra were obtained at 15 K at X-band and on field positions corresponding to the maxima of the field swept spectra with pulse lengths of 16 and 32 ns for π/2 and π pulses, respectively. *τ* was set to a ^1^H blind spot (∼290 ns) as determined from the 3-pulse ESEEM, and the initial *t*_1_ and *t*_2_ were set to 56 ns, using a 4-step phase cycle. Data were processed and analysed using HYSCOREAN,^29^ employing Hamming apodisation, 3^rd^ order polynomial background correction, and diagonal and anti-diagonal spectral symmetrisation, keeping a similar amount of noise for each spectrum by adjusting the minimum contour level percentage accordingly.

### 2.8. Relaxation-induced dipolar modulation enhancement (RIDME; deuterated sample)

Relaxation time measurements were conducted at 30 K at Q-band frequencies (34 GHz) at the field position corresponding to the maximum of the Cu^2+^ field-sweep spectrum for the sample with 250 µM protein and 2 molar equivalents of Cu^2+^. Transverse dephasing time constant *T*_m_ (corresponding to *T*_2_ and additional dephasing mechanisms) was determined from the 2-pulse electron–spin echo decay experiments recorded with a starting *τ* of 200 ns using pulse lengths of 16 and 32 ns for π/2 and π pulses respectively, fitting a stretched exponential decay function to the data. Longitudinal relaxation time constants *T*_1_ were estimated from the 3-pulse inversion recovery measurements recorded with a *τ* of 800 ns, an 8 ns inversion pulse and 16 and 32 ns observer pulses (π/2 and π pulses respectively), fitting a biexponential model to the data.

With respect to the width of the Cu^2+^ spectrum (∼5 GHz), the narrow bandwidth of the pulses (100–300 MHz) does not allow homogeneous sampling of all Cu^2+^ orientations from a single magnetic field position. Therefore the 5-pulse RIDME experiments^30^ were performed at three individual field positions,^31^ an offset of −100, −600 and −800 G respectively from the field position corresponding to the maximum of the Cu^2+^ field-sweep spectrum, to average the orientation selection effect, using the pulse sequence (π/2 − *τ*_1_ − π − (*τ*_1_ + *t*) − π/2 − *T*_mix_ − π/2 − (*τ*_2_ − *t*) − π − *τ*_2_ − echo) with 8-step phase cycling. A *τ*_1_ of 400 ns and a *τ*_2_ of 2500 ns, with the shot repetition time (SRT) of 400 µs was used. Measurements were recorded with a mixing time of 0.7 × *T*_1_ (longitudinal relaxation time, assessed from the biexponential approximation). The data were analysed using ComparativeDEERAnalyzer,^32,33^ (CDA) 2.0 within DeerAnanlysis2022,^34^ for the individual field positions and the summed time trace.

### 2.9. Simulations of distance distributions

The distances between the Cu^2+^ atoms in the structure reported herein were measured in PyMOL.^35^ Gaussian distance distributions were then simulated using the Python-based DeerLab program,^32^ by selecting a combination of three atoms with equal amplitudes and a standard deviation of 0.1 nm.

## 3. Results and discussion

### 3.1. Structural characterisation of Cu^2+^ complexes with ESA

**Fig. 1:**
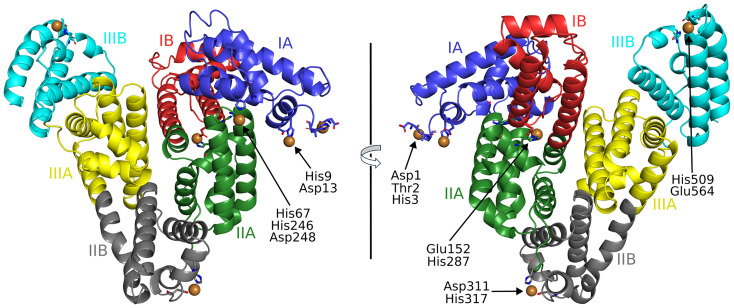
Locations of Cu^2+^-binding sites on ESA. Cu^2+^ ions are shown as brown spheres. Residues belonging to the first coordination spheres are shown as sticks and labelled, each on only one panel. Domains are labelled with Roman numerals (I, II, III) and subdomains with letters (*e.g.*, IB), with each subdomain shown in a different colour (as indicated).

The full locations and compositions of Cu^2+^-binding sites on serum albumin have not previously been characterised. Here we obtained an ESA structure with Cu^2+^ bound (PDB ID: 9zmd). A summary of the crystal structure with the metal binding sites labelled is presented in Fig. 1. The ESA crystal structure presented belonged to the *P*61 space group. The structure contained a single protein chain in the asymmetric unit. The structure adopted the characteristic “heart-shaped” albumin fold and conserved 17 disulfide bridge structure found in other albumin structures.^11–13,36,37^ The slightly elevated *R*_free_–*R*_work_ gap is consistent with values commonly observed for albumin structures of comparable resolution and reflects the intrinsic flexibility of albumin (Fig. S2). In addition, the density was sufficient to allow the ATCUN residues to be modelled, which is not always the case in albumin structures due to the inherent flexibility of this region.

To examine this, we identified 320 albumin molecules in the PDB, including those from crystal structures that contain multiple copies of the protein in the asymmetric unit. Analysis of these structures revealed substantial flexibility in the N-terminal region, as only 8% have the first residue modelled, while 18% contain a modelled second residue (Fig. S3). Crystal packing can strongly influence this region and fortunately, in our Cu^2+^–ESA structure, the ATCUN motif coordinating Cu^2+^ is stabilised by a symmetry-related protein located 3.4 Å away. This interaction likely contributes to the defined electron density observed for the first residues (Fig. S4A). A similar effect was observed in our earlier study of the Co^2+^-HSA crystal structure (PDB ID: 8ew4). In that case, contacts between the N-terminus and a symmetry-related molecule, combined with Co^2+^ coordination, led to improved local electron-density map quality (Fig. S4B). In contrast, other albumin structures, such as PDB ID: 1bj5, which lack nearby symmetry contacts and instead contain a solvent-filled cavity near the N-terminal region, show insufficient electron density to model the first two residues (Fig. S4C).

The intrinsic flexibility of the N-terminal region makes co-crystallisation essential for capturing Cu^2+^ binding, as the ATCUN motif (comprising the first three residues) must adopt a specific conformation to coordinate the metal. This contrasts with the more pre-organised site A and site B involved in Zn^2+^,^11^ or Co^2+^,^12^ binding, where soaking can be successfully applied to pre-formed albumin crystals. For example, in a Co^2+^–HSA crystal structure (PDB ID: 8ew4; Fig. S4B),^12^ the local packing around the N-terminus is relatively tight due to several interactions with a symmetry-related protein. If this crystal form were used for Cu^2+^ soaking, it would likely not allow the N-terminus to rearrange and form the canonical ATCUN coordination mode observed in the presented Cu^2+^–ESA structure.

### 3.2. Structures of Cu^2+^-binding sites in ESA

**Fig. 2:**
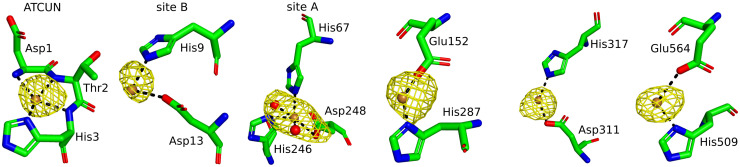
Binding sites and anomalous signals (RMSD = 3.5) in the ESA structure (PDB ID: 9zmd). Brown spheres represent Cu^2+^ ions, and red spheres represent water molecules. Binding sites are listed in numerical order according to residue position.

The anomalous map peaks indicated six locations for bound Cu^2+^, where coordinating residues were sufficiently ordered to be modelled (Fig. 2). Representative 2*F*_o_–*F*_c_ electron density maps for all Cu^2+^ coordination sites are shown in Fig. S5. As mentioned, one of these sites corresponds to the ATCUN motif (Asp1–Thr2–His3), where the Cu^2+^ ion adopts a square-planar coordination geometry. This arrangement closely resembles that reported for the high-resolution Cu^2+^–DAHK peptide complex (Fig. S6A),^38^ in which Cu^2+^ is coordinated by the N-terminal amine, two deprotonated amide nitrogens, and the imidazole nitrogen of histidine. A structural superposition of the two motifs confirms the conservation of this canonical ATCUN geometry (Fig. S6B). It is noteworthy that the term ATCUN was originally derived from studies on albumins, particularly HSA and BSA,^5–7,39^ making albumin the prototypical ATCUN-containing protein. While metal binding at ATCUN sites in other proteins have been reported previously, mainly for Ni^2+^ (Table S2 and Fig. S6C) to our knowledge, this is only the second structure in the PDB where Cu^2+^-coordination at such a site has been observed. The first was in Cu-carbonic anhydrase-II (Cu-CA-II; PDB ID: 6pdv), where a characteristic planar geometry was found.^40^ It is thought that this site in Cu-CA-II may serve in allowing transfer of electrons to the copper active site.^41^

Cu^2+^ binding at site B of ESA was also observed with N- and O-coordination from His9 and Asp13 sidechains but other ligands, likely from water molecules, were not visible. Site A residues also participated in metal coordination, with Cu^2+^ forming a site involving sidechain groups from His67, His246, Asp248 and two water molecules. In agreement with our previous study, which involved an examination of Cu^2+^-binding sites in HSA using mutagenesis in combination with EPR-based approaches, Cu^2+^ was found to coordinate to an imidazole nitrogen of the conserved His287 residue (His288 in HSA), in addition to a sidechain oxygen from Glu152. Cu^2+^ additionally bound at distinct sites in domains II and III. The first of these involved Asp211 (sidechain oxygen) and His317 (sidechain nitrogen) and the second, His509 (sidechain nitrogen) and Glu564 (sidechain oxygen), respectively. As with site B, it was not possible to observe additional water ligands in the coordination sphere.

Several of the residues involved in Cu^2+^ coordination in ESA have also been reported to bind other metal ions or metal-containing compounds in albumin crystal structures (Table S1). The ATCUN site is, to date, unique to Cu^2+^ coordination, whereas site A is a versatile metal-binding site that also accommodates Zn^2+^, Co^2+^, and Pt^2+^ (the latter as part of the anti-cancer drug cisplatin). Site B has been reported to bind Co^2+^, and the conserved His287/His288 site has been shown to coordinate cisplatin and Fe-based metal compounds. Pt^2+^ from cisplatin has also been observed to engage additional histidine residues in albumin structures that are not involved in Cu^2+^ binding. These differences likely reflect distinct coordination preferences: Pt^2+^ is a soft Lewis acid with strong affinity for histidine and sulfur donors, whereas Cu^2+^ is a borderline Lewis acid that typically requires a multidentate coordination environment for stable binding.

In contrast to the present structure, previously reported crystal structures of HSA in complex with Cu^2+^–thiosemicarbazone and related metal containing compounds show the metal ion coordinated at His146 and/or His242 (Table S1),^14^ which correspond to ligand-binding pockets rather than physiological metal ion sites. In these complexes, the Cu(ii) centre remains pre-coordinated by the organic ligand scaffold, which dictates recognition of, and interactions with, the albumin ligand-binding pocket, with the protein contributing only a single anchoring residue that coordinates the Cu^2+^ ion. By comparison, in the present structure free Cu^2+^ is coordinated by multiple protein donors at each binding site. Consistent with this fundamental difference in coordination chemistry, we did not observe any evidence for free Cu^2+^ binding at His146 or His242 in ESA.

### 3.3. Probing Cu^2+^ binding to ESA using EPR-based approaches

**Fig. 3:**
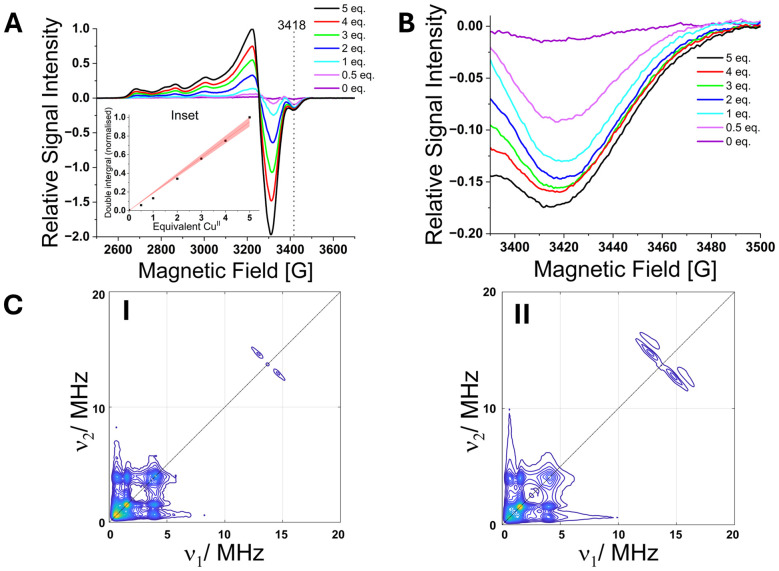
(A) Selected individual CW EPR spectra normalised and overlaid. Inset shows double integration of Cu^2+^, normalised. (B) The distinct spectral component for the ESA pseudo-titration series at ∼3418 G corresponding to a lower *g* value for the first (high affinity) binding site. (C) Representative (+,+) HYSCORE spectra for ESA with 0.5 (I) and 5.0 (II) molar equivalents of Cu^2+^, obtained at X-band frequencies (9.5 GHz) and on the maximum of the field-swept spectra.

Continuous-wave (CW) EPR spectroscopy was employed to investigate high-affinity Cu^2+^ binding sites of ESA. Double integration of the spectra revealed a linear increase in the Cu^2+^ signal from 0.5 up to 5.0 molar equivalents of Cu^2+^ added (Fig. 3A). This linearity indicates that all added Cu^2+^ was EPR active, with no evidence for antiferromagnetically coupled dimeric type III-like Cu^2+^ species. Inspection of individual CW EPR spectra showed the emergence of a distinct spectral feature at approximately 3418 G upon addition of just 0.5 molar equivalents of Cu^2+^. This signal is attributed to the highest-affinity Cu^2+^ binding site, likely the N-terminal ATCUN motif, which is known to exhibit a lower *g*_∥_ and a large *A*_∥_ (here simulated with *g*_∥_ = 2.198 and *A*_∥_ = 607 MHz; Table S3 and Fig. S7) compared to other sites consistent with square-planar copper coordination.^39^ Notably, this feature did not increase further upon addition of more than 1 equivalent of Cu^2+^, suggesting that the high-affinity site was fully occupied at this point (Fig. 3B), similar to the situation in HSA.^10^ Further analysis of the spectral line shapes revealed the presence of multiple Cu^2+^ species already from 0.5–1 molar equivalents of Cu^2+^ onwards, consistent with the coexistence of several coordination environments (Fig. S8, S9 and Table S4). These included nitrogen donors such as histidine and imidazole (potentially from Tris buffer), as well as oxygen donors, consistent with the sites observed in the X-ray crystal structure.^42–44^ Thus, CW EPR data of the pseudo-titration series suggested the presence of multiple Cu^2+^-binding sites besides the ATCUN motif of similar high affinity, while the highest-affinity ATCUN site seemed to be partly pre-occupied, potentially through some diamagnetic metal (*e.g.*, Ni^2+^) being co-purified, which would explain the early appearance of additional sites being occupied by Cu^2+^.

To probe the involvement of specific ligands, 3-pulse ESEEM experiments were conducted. The resulting spectra displayed characteristic nuclear quadrupole interaction (NQI) signals between ∼0.5–2 MHz, along with a broader double quantum (DQ) transition at ∼4 MHz (Fig. S10). These features are consistent with remote ^14^N nuclei in the imidazole rings of histidine residues, confirming their participation in Cu^2+^ coordination. These findings were corroborated by two-dimensional hyperfine sublevel correlation (HYSCORE) spectroscopy. Both samples with 250 µM ESA and 0.5 and 5 molar equivalents of Cu^2+^, respectively, exhibited similar combination peaks for weakly coupled ^14^N nuclei in the range of ∼0.5–4 MHz (Fig. 3C and S11). Additionally, the HYSCORE data revealed an increase in water-coordinated Cu^2+^ species with higher Cu^2+^ loading, as shown in the increase in intensity of the splitting in the signal at ∼14 MHz.^10^ This suggests the absence of an apically bound water molecule at the ATCUN site, as seen for HSA.^11^

**Fig. 4:**
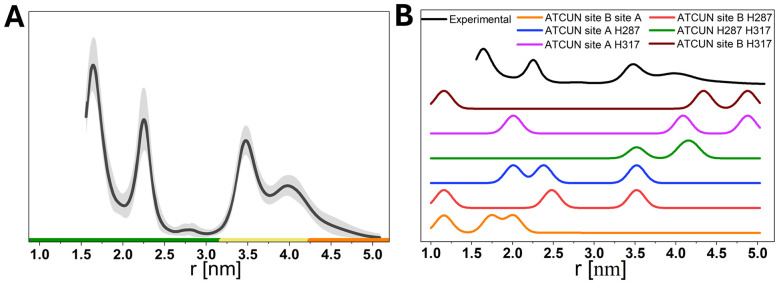
(A) PDS (RIDME) distance distribution for ESA with 2 molar equivalents of Cu^2+^. Distance distributions are given as mean (black line) with 95% confidence estimate (grey shaded area). Colour bars represent reliability ranges (green: shape reliable; yellow: mean and width reliable; orange: mean reliable). (B) Stacked comparison of experimental distance distribution (black) with simulated distance distributions for ESA at 2 molar equivalents of Cu^2+^ binding at different histidine sites. Each coloured trace represents a distinct Cu^2+^ occupancy configuration involving ATCUN site, site B, site A, His287 and His317.

The experimentally obtained pulsed dipolar EPR spectroscopy (PDS) distance distribution (from RIDME experiments; Fig. 4A) exhibits several distinct peaks. We extracted all pairwise copper distances from the structure and tabulated them (Table S5). We further simulated distance distributions based on a model of simultaneous occupation of 3 sites (Fig. 4B). The distance between the ATCUN site and site B is too short for reliable identification by PDS and might contribute to the lower edge experimental distance probability between 1.5 and 2.0 nm (Fig. 4B black). Assuming the ATCUN site to be populated as well as sites A and B (Fig. 4B orange) it leads to additional contributions around 2.0 nm but cannot explain experimental distance probability at 3.5 nm and 4 nm. Indeed, involving the distance between ATCUN site and Cu^2+^ at H287 matches 3.5 nm very well (Fig. 4B blue, red, green). This agrees with the involvement of H288 in Cu^2+^ binding in HSA.^10^

The appearance of the broader signal around 4 nm could be reproduced by the occupation of the distal H317 site. Distances between the site at H317 and the ATCUN site, sites A and B and the site at H287 lie all between 4.0 and 4.9 nm (Fig. 4B green, magenta, brown). Notably, the ATCUN site and site B appear to coexist in most configurations, as their combined occupancy defines the short to mid-range distance distributions. This is again similar to the situation in HSA where His9 was identified as a Cu^2+^ binding site using EPR, and the involvement of His9 at the ATCUN site at low Cu^2+^ load was hypothesised but not unequivocally demonstrated.^10^ Finally, the site at His509 cannot be evaluated by this data as distances to all other sites lie between 4.5 and 7.6 nm at the edge or outside the distance resolution achieved here. Interestingly, a knockout of His67 in HSA did not alter the experimental RIDME PDS distance distribution significantly.^10^ This may be because site A has two histidine ligands (His67 and His246/His247 in ESA/HSA) and some occupation may be retained if one is knocked out. The present data are not sufficient to rule out the occupation of any of the sites, but it clearly hints at (at least) partial occupation of the sites at His287 and even His317 at 2 molar equivalents of copper(ii).

## 4. Conclusions

In summary, the X-ray crystal structure of ESA bound to Cu^2+^ is presented, revealing the high-affinity ATCUN site with square-planar geometry. This was fortuitously enabled by a symmetry-related interaction that stabilised the flexible N-terminal region. Five additional Cu^2+^ sites were identified, including sites A and B and other sites involving histidine ligands. CW EPR data supported the presence of a square-planar coordination at the ATCUN site. ESEEM and HYSCORE enabled detection of ^14^N signals and quadrupole interactions, indicating histidine involvement in Cu^2+^-binding and increased water coordination at higher Cu^2+^ loading. RIDME distance distributions and simulations revealed the simultaneous occupation of multiple sites, with strong evidence for ATCUN and site B binding with partial engagement of sites involving distal histidine residues. These findings support a multi-site Cu^2+^ binding model that is consistent with prior albumin studies.

## Author contributions

A. J. S., B. E. B. and W. M. designed the research; T. R. and B. L. prepared materials; K. B. H., M. G., V. B., K. A., A. C., J. S. and B. E. B. performed experiments (K. B. H., M. G., V. B. and J. S. carried out X-ray crystallography and K. A., A. C., and B. E. B. performed EPR-based experiments); K. B. H., M. G., A. P., K. A., A. C., J. S., C. A. B., A. J. S., B. E. B. and W. M. analysed and interpreted the results; K. B. H., M. G., K. A., A. J. S., B. E. B. and W. M. wrote the manuscript; all authors reviewed and approved the final version.

## Conflicts of interest

WM has been involved in the development of state-of-the-art software, data management and mining tools; some of these have been commercialised by HKL Research and are mentioned in the paper. WM is the co-founder of HKL Research and a member of the board. The authors have no other relevant affiliations or financial involvement with any organisation or entity with a financial interest in or financial conflict with the subject matter or materials discussed in the manuscript apart from those disclosed.

## Supplementary Material

QI-013-D6QI00150E-s001

## Data Availability

X-ray structural coordinates were deposited in the RCSB Protein Data Bank with the PDB ID: 9zmd. The corresponding raw X-ray diffraction data have been deposited in the Integrated Resource for Reproducibility in Macromolecular Crystallography (IRRMC) and are available at https://doi.org/10.18430/M39ZMD. EPR spectroscopic data are available at https://doi.org/10.17630/5b97443d-4fed-4b38-b33c-c8915da4fefe. Supplementary information (SI): raw data and additional data. See DOI: https://doi.org/10.1039/d6qi00150e.
