# Supplementary material for: Crystallographic and EPR-based characterisation of Cu2+-binding to serum albumin: ATCUN coordination and additional sites
Source: Inorg Chem Front. 2026 Mar 12;13(9):3750–8. doi: 10.1039/d6qi00150e (PMC13001715; doi:10.1039/d6qi00150e)
Supplement: QI-013-D6QI00150E-s001 [file QI-013-D6QI00150E-s001.pdf]

Supplementary Information (SI) for Inorganic Chemistry Frontiers.
This journal is © the Partner Organisations 2026

Supplementary Information

Crystallographic and EPR-based characterisation of Cu^{2+}-binding to serum albumin: ATCUN coordination and additional sites

Michal Gucwa, Katarzyna B. Handing, Vanessa Bijak, Katrin Ackermann, Aisika Chakraborty, Anastasiya Pautarak, Timothy Redpath, Boyang Lin, Joanna Slawek, Claudia A. Blindauer, Alan J. Stewart, Bela E. Bode, Wladek Minor

Table S1 Crystal structures of serum albumins with bound metal species deposited in the Protein Data Bank.

Figure S1 Resolution distribution of serum albumin crystal structures deposited in the Protein Data Bank.

Figure S2 Distribution of differences between Rfree and Rwork values for structures deposited in the Protein Data Bank and for serum albumin structures specifically.

Figure S3 Distribution of the first modeled residue in albumin structures deposited in the Protein Data Bank.

Figure S4 Influence of symmetry mate interactions on modeling the N-terminal residues.

Figure S5 2Fo–Fc electron density maps around Cu^{2+} coordination sites in ESA, showing ATCUN, site B, site A, and addional bindig sites.

Figure S6 Structural details of the ATCUN motif.

Table S2 List of Protein Data Bank structures containing an ATCUN motif with a bound metal ion.

Table S3 List of CW EPR simulation parameters for ESA and 0.5 molar equivalents of Cu^{2+}.

Figure S7 Experimental and simulated CW spectra for the ESA and 0.5 molar equivalents of Cu^{2+}.

Figure S8 Individual CW spectra of the ESA pseudo-titration series with 0 to 5 molar equivalents of Cu^{2+} added.

Figure S9 Experimental and simulated CW spectra for the ESA and 2 molar equivalents of Cu^{2+}.

Table S4 List of CW EPR simulation parameters for ESA and 2 molar equivalents of Cu^{2+}.

Figure S10 Individual ESEEM spectra of the pseudo-titration series with 0.5 and 5 molar equivalents of Cu^{2+} added.

Figure S11 Individual HYSCORE spectra of the pseudo-titration series with 0.5 and 5 molar equivalents of Cu^{2+} added.

Table S5 List of distances between the Cu^{2+} bound at different sites.

Table S1 Crystal structures of serum albumins with bound metal species deposited in the Protein Data Bank (PDB). The table summarises the ligand residue name, metal ion, PDB identifier, classification of the metal species (free metal ion or metal complex), and the key albumin residues involved in metal coordination.

Ligand Residue Name	Metal Ion	PDB ID	Comment on Metal Ion	Key Binding Residues
CU	Cu	9zmd	Free Metal Ion	Asp1, Thr2, His3, His9, Asp13, His67, Glu152, His246, Asp248, His287,

				Asp311, His317
ZN	Zn	5iix, 5iiu, 5ije, 5iih, 5ij5, 5ijf	Free Metal Ion	His67, His246, Asp248
CO	Co	8ew7, 7mbl, 8ew4, 8ey5	Free Metal Ion	His9, Asp13, His67, Asp248
RU	Ru	8k1y, 7dl4, 8h0o, 5ifo	Free Metal Ion/Cl-	His146, His242, Arg348, Arg485
CA	Ca	6qs9, 3v03, 4jk4	Free Metal Ion	Glu6, Asp13, Ser109, Asp111, Glu243, Asp248, Glu251, Asp254, Asp258
NA	Na	6hn0, 8bsg	Free Metal Ion	His251, Asp255
AU	Au	6rjv	Free Metal Ion	Cys34
K	K	6a7p	Free Metal Ion	Free
YT3	Y	7a9c	Free Metal Ion	Glu131, Glu252, Glu244
MG	Mg	6rjv	Free Metal Ion	Ser109, Asp111, Asp248
FE	Fe	7wlf	Free Metal Ion	His288, His128
PT	Pt	9hnb	Free Metal Ion	His146, Met329, Met298
CPT	Pt	4s1y, 7woj, 7wok	Cis-platin	His67, His105, His128, His146, His247, Asp249, His288, His338, His440, Met298, Met329, Met548
HEM	Fe	1n5u, 1o9x	HEM	Tyr161
7GE	Ru	5giy	[RuCl5(ind)]2	His146, His242
M6O	Al	6xv0	Lauric Acid Compound	Arg197
M6O	Mo	6xv0	Lauric Acid Compound	Arg197
6WF	Fe	5gix	Fe(III)-Thiosemicarbazone complex	His242
7Q8	In	7wz9	In(III)-Thiosemicarbazone complex	His146
8ZR	Cu	5yb1	Cu(II)-Thiosemicarbazone complex	His146
A1D6U	Cu	8yg7	Cu(II)-Thiosemicarbazone complex	His146
E5O	Cu	6l4k	Cu(II)-Thiosemicarbazone	His242

			complex	
IC4	Cu	7y2d	Cu(II)-Thiosemicarbazone complex	His146, His242
J2O	Au	7eek	Au(III)-Thiosemicarbazone complex	His146
U5U	Pd	8j8e	Pd(II)-Thiosemicarbazone complex	His242
ZJZ	Pt	8ism	Pt(II)-Thiosemicarbazone complex	His242

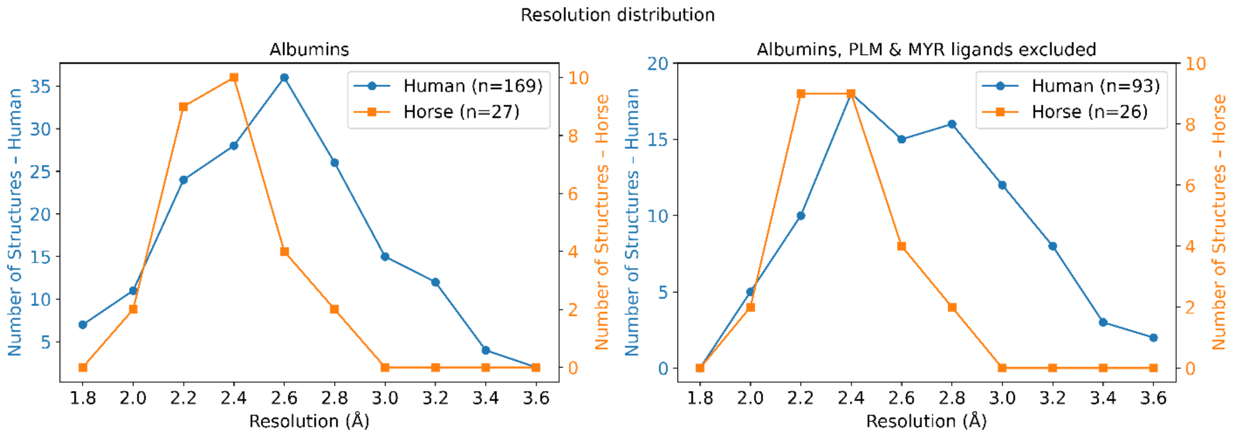

Figure S1 Resolution distribution of serum albumin crystal structures deposited in the Protein Data Bank (PDB). Left panel: structures of human serum albumin (HSA) and equine serum albumin (ESA). Right panel: the same dataset after exclusion of structures containing palmitic acid (PLM) and myristic acid (MYR), two commonly used fatty-acid ligands employed to stabilisze albumin for crystallization. We acknowledge the simplicity of this analysis, as albumin structures may contain other bound fatty acids or ligands that also influence protein rigidity and crystallisability; however, PLM and MYR represent the most frequently used albumin stabilising ligands in the PDB and therefore provide a reasonable first-order comparison of intrinsic crystalliszation behavior between HSA and ESA.

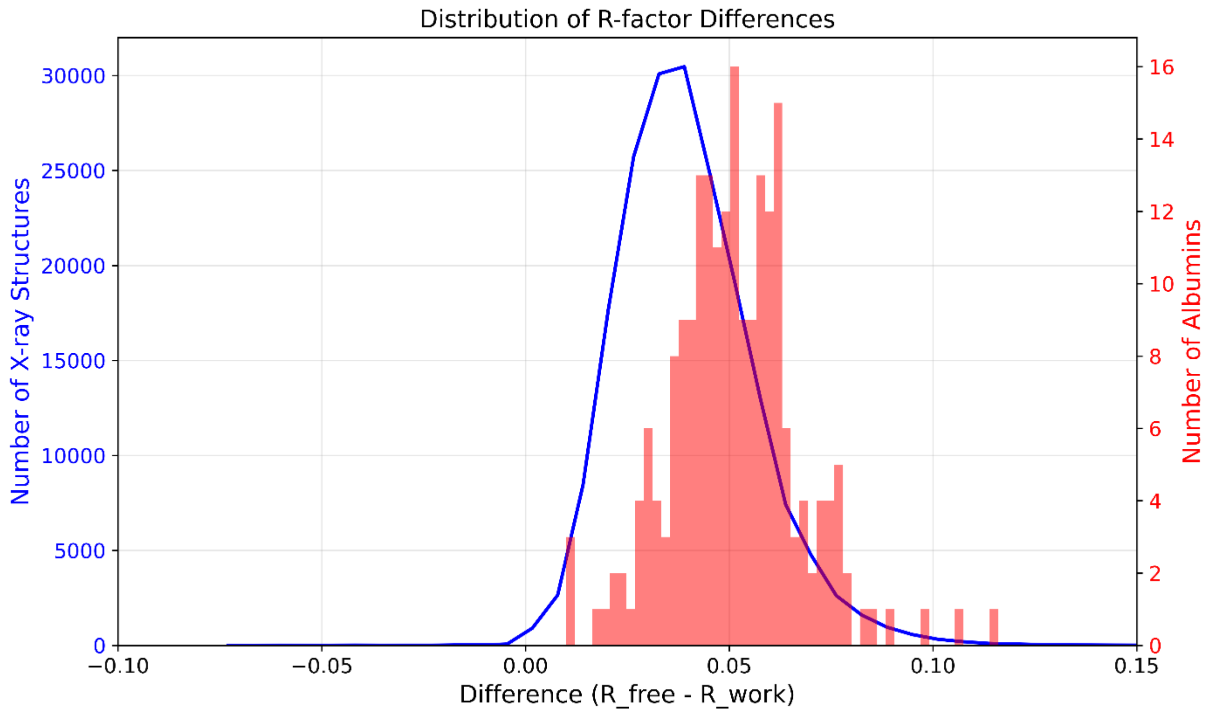

Figure S2 Distribution of differences between Rfree and Rwork values for structures deposited in the Protein Data Bank (blue curve, all structures) and for serum albumin structures specifically (red histogram). The x-axis shows the Rfree–Rwork difference.

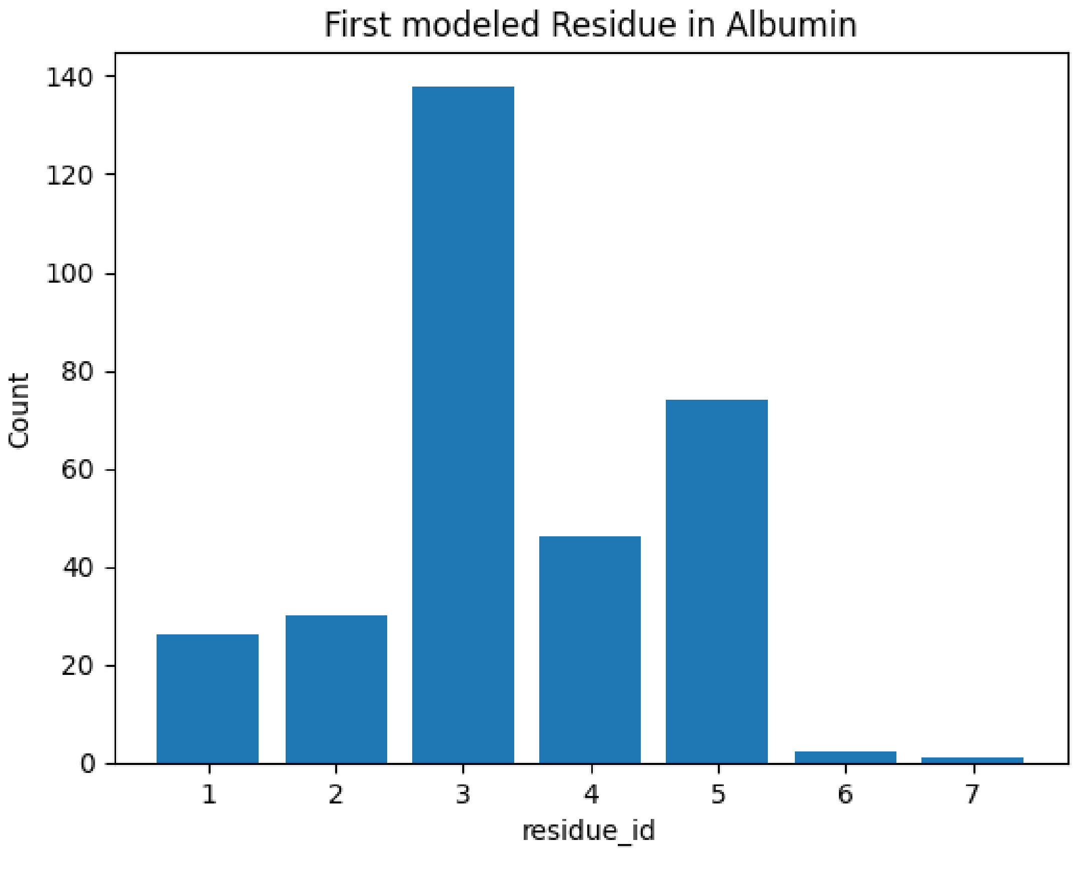

Figure S3. Distribution of the first modeled residue in albumin structures deposited in the Protein Data Bank. The histogram shows the frequency of the first residue modeled in 320 albumin chains, as some PDB entries contain multiple molecules in the asymmetric unit.

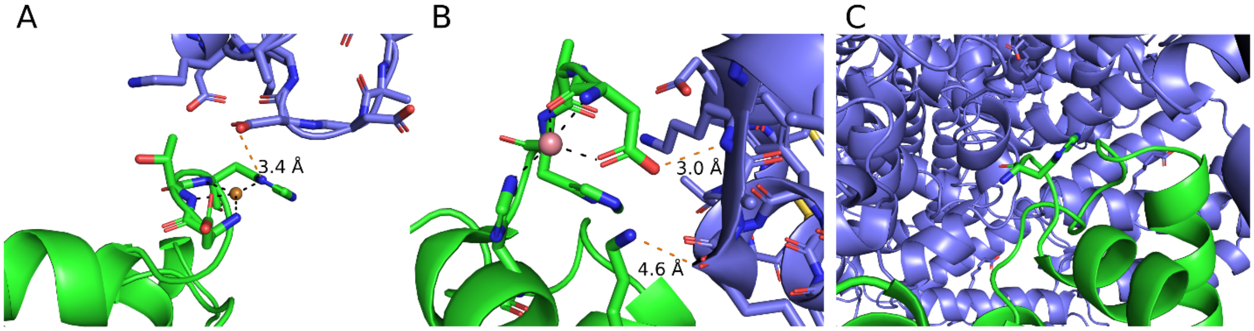

Figure S4. Influence of symmetry mate interactions on modeling the N-terminal residues. The reference molecule is shown in green, and the symmetry mate in blue. **A**) Cu^{2+}–ESA structure (this work), showing the first three residues of the ATCUN motif coordinated to a Cu^{2+} ion; the closest atom from the symmetry mate is 3.4 Å away. **B**) Co^{2+}–HSA structure (PDB ID: 8ew4), with coordinated Co^{2+} and a few interactions between the N-terminus and the symmetry mate. **C**) HSA structure (PDB ID: 1bj5), where modeling begins at residue 3 due to the absence of close crystal contacts and the presence of a solvent-filled cavity near the N-terminus, leading to increased flexibility and insufficient electron density to model the first two residues.

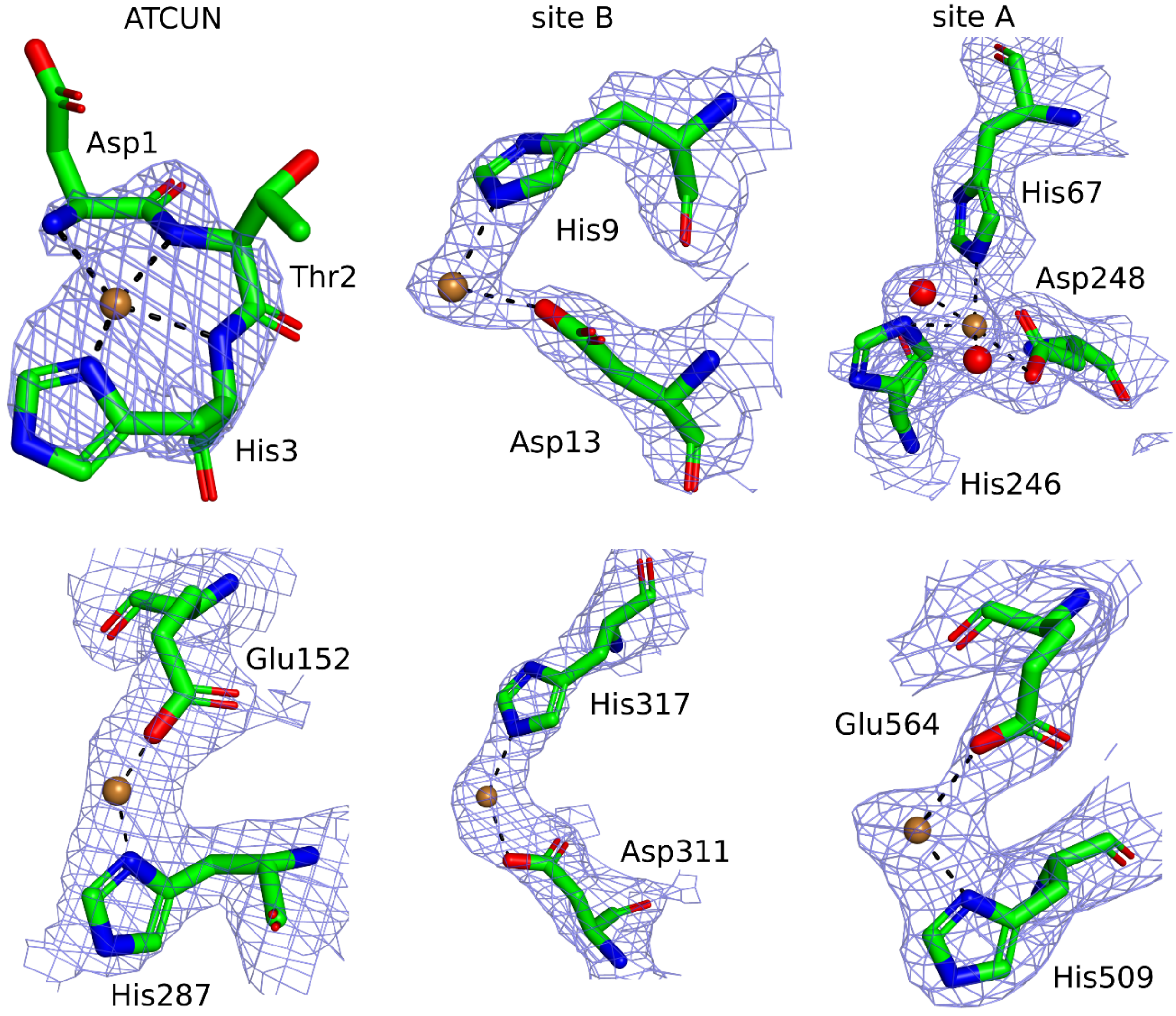

Figure S5 2Fo–Fc electron density maps (contoured at 1σ) around Cu^{2+} coordination sites in ESA (PDB ID: 9zmd), showing ATCUN, site B, site A, and additional binding sites.

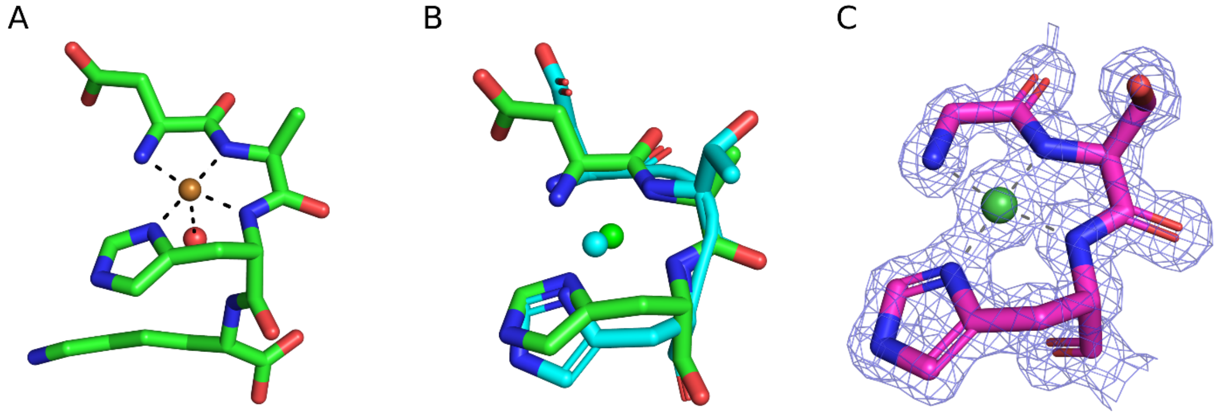

Figure S6 Structural details of the ATCUN motif. **A**) High-resolution crystal structure of the Cu(II)–DAHK complex (Cambridge Structural Database ID: 809109), illustrating the canonical ATCUN coordination geometry. The Cu^{2+} ion adopts a tetragonally elongated square-pyramidal environment with four short equatorial Cu–N bonds (1.9–2.0 Å) and a longer axial Cu–O(water) interaction (~2.6 Å). Cu^{2+} is shown as a brown sphere and the axial water molecule in red. **B**) Superposition of the ATCUN motifs from the Cu(II)–DAHK complex (green) and the Cu–ESA structure reported here (PDB ID: 9zmd; cyan), highlighting the conserved coordination geometry. **C**) Example of a well-resolved ATCUN site from the PDB (PDB ID: 4rtz; resolution 0.979 Å), showing clearly defined 2Fo–Fc electron density around the coordinating residues. Panels A and C serve as high-resolution references for comparison with the moderate-resolution Cu–ESA structure presented in this work.

Table S2 List of Protein Data Bank structures containing an ATCUN motif with a bound metal ion. The table includes the PDB ID, identity of the central metal ion, and crystallographic resolution. The Central Element column specifies the metal site in the format

element:chain:residue number. Columns are duplicated to conserve space. A total of 64 ATCUN motifs were identified: 59 with Ni^{2+}, 2 with Co^{2+}, 1 with Cu^{2+}, 1 with Zn^{2+}, and 1 with Ca^{2+}.

PDB ID	Central Element	Resolution	PDB ID	Central Element	Resolution
4b1u	Ca:M:1163	2	4i75	Ni:A:404	1.8
4d07	Co:A:1090	1.85	6deh	Ni:A:402	1.8
4aq4	Co:A:1419	1.8	6deh	Ni:B:401	1.8
6pdv	Cu:A:302	1.23	2rj2	Ni:A:501	1.7
1ox4	Ni:A:902	2.5	4i74	Ni:A:405	1.68
1ox4	Ni:B:901	2.5	8qov	Ni:A:501	1.6
1ox5	Ni:A:902	2.5	7cxz	Ni:A:303	1.561
2rab	Ni:A:468	2.5	4jz4	Ni:A:201	1.56
2rab	Ni:B:468	2.5	4jz4	Ni:B:201	1.56
6s5i	Ni:A:201	2.45	4c24	Ni:A:302	1.5
7u0v	Ni:A:201	2.45	8cwr	Ni:B:603	1.5
1ox6	Ni:A:902	2.4	6r54	Ni:A:303	1.417
1ox6	Ni:B:901	2.4	8qou	Ni:A:203	1.4
3rdh	Ni:A:247	2.39	8qou	Ni:B:401	1.4
3rdh	Ni:B:247	2.39	8cwt	Ni:B:603	1.35
3rdh	Ni:C:247	2.39	8cwt	Ni:D:601	1.35
3rdh	Ni:D:247	2.39	8cwt	Ni:F:602	1.35
3um9	Ni:A:230	2.19	4rtx	Ni:A:201	1.32
3um9	Ni:B:230	2.19	4rtx	Ni:B:201	1.32
4i73	Ni:A:406	2.18	4rtx	Ni:C:201	1.32
4i73	Ni:D:404	2.18	4rtx	Ni:D:201	1.32
1jvn	Ni:A:902	2.1	6xx5	Ni:A:201	1.3
1jvn	Ni:B:901	2.1	4i71	Ni:A:404	1.28
2r9z	Ni:A:468	2.1	3zqw	Ni:A:1156	1.07
2r9z	Ni:B:468	2.1	6r4z	Ni:A:302	1.052
6a6f	Ni:A:207	2.1	6xx4	Ni:A:201	1.05
6a6f	Ni:B:210	2.1	4omo	Ni:A:201	1.04
4i72	Ni:A:405	2.05	4omo	Ni:B:201	1.04
4i72	Ni:B:405	2.05	3zuc	Ni:A:1156	1.001
4c25	Ni:A:302	2.03	4rtz	Ni:A:201	0.979
6r58	Ni:D:302	1.9	1xmk	Ni:A:398	0.97
3rva	Ni:A:455	1.8	1ro5	Zn:A:405	2.3

Table S3 CW EPR simulation parameters for the spectrum shown in ***Figure S7*** below. The A-values are provided in MHz and rounded, values for the line width are provided in mT.

Centre	Cu^{II}
g_{11}	2.061
g_{22}	2.061
g_{33}	2.198

A_{11}	31.5
A_{22}	31.5
A_{33}	607
Linewidth	6

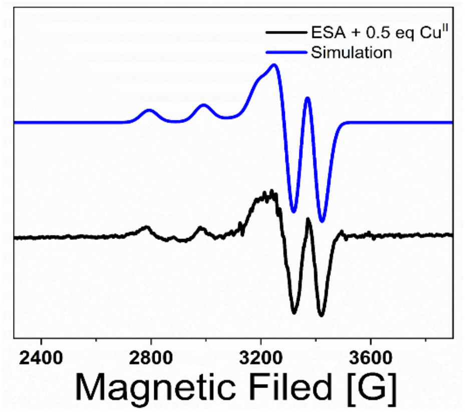

Figure S7 Experimental (black) and simulated (blue) CW spectra for the ESA and 0.5 molar equivalents of Cu^{2+}. Corresponding simulation parameters are provided above in **Table S3**.

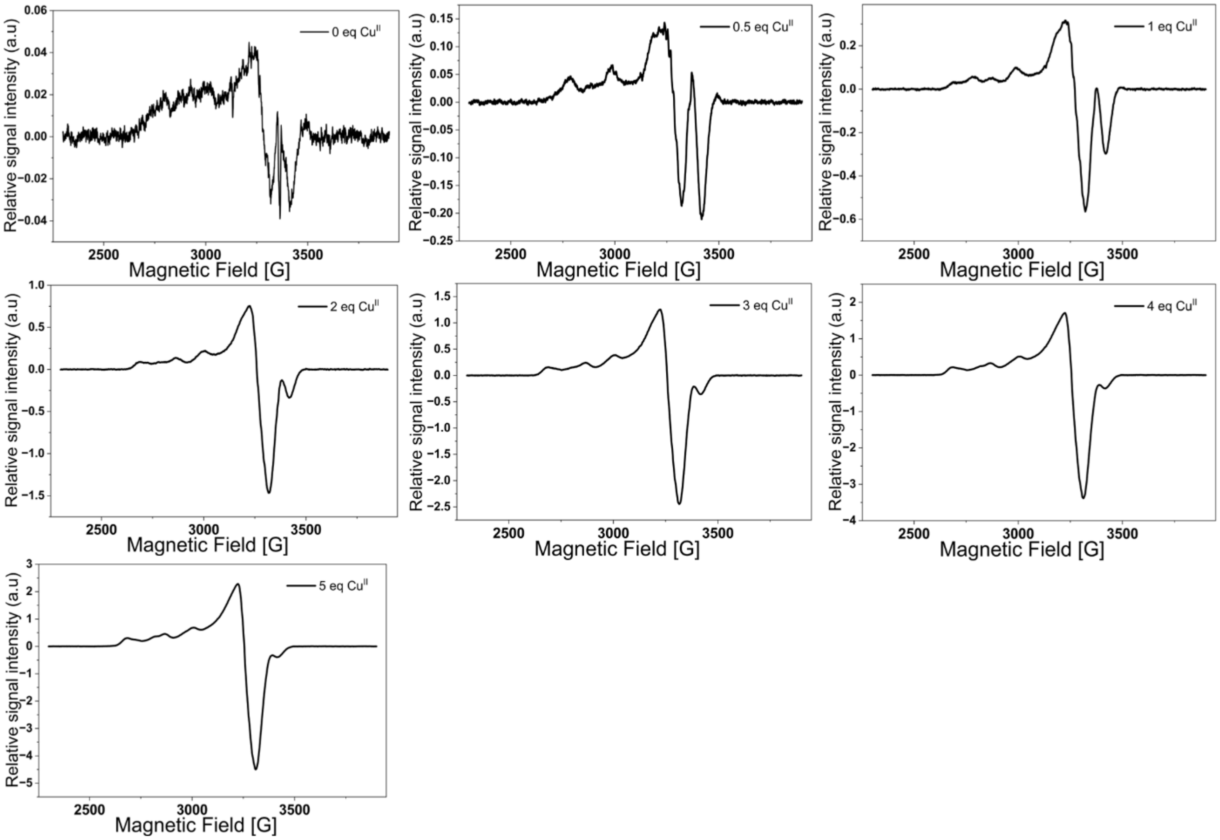

Figure S8 Individual CW EPR spectra of the ESA pseudo-titration series with 0 to 5 molar equivalents of Cu^{2+} added.

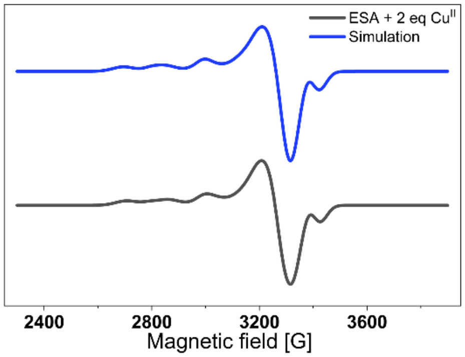

Figure S9. Experimental (black) and simulated (blue) CW spectra for the ESA and 2 molar equivalents of Cu^{2+}. Corresponding simulation parameters are provided below in ***Table S4*****.**

Table S4 CW EPR simulation parameters for the spectrum shown in **Figure S9** above. The A-values are provided in MHz and rounded (a respective A-strain is provided in brackets), values for the line width are provided in mT.

Centre	Cu^{II} (1)	Cu^{II} (2)
g_{11}	2.075	2.046

g_{22}	2.075	2.072
g_{33}	2.320	2.194
A_{11}	16 (51)	7.95 (75)
A_{22}	15 (50)	7.88 (98)
A_{33}	483 (50)	595 (50)
Linewidth	7.71	4.95
Weight	0.77	0.23

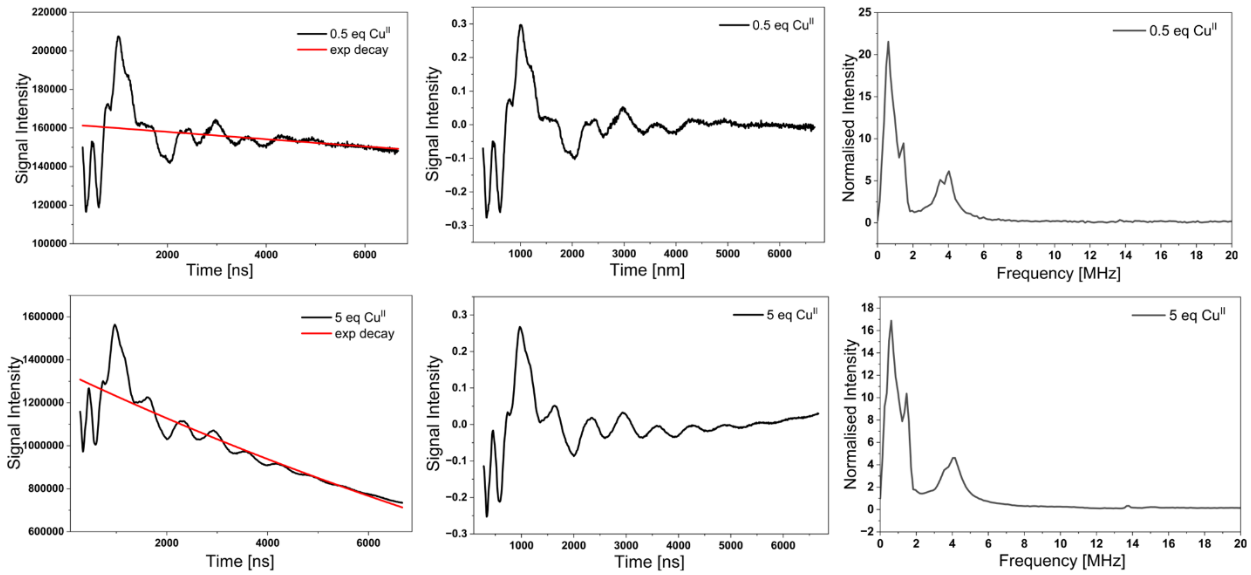

Figure S10. Individual ESEEM spectra of the pseudo-titration series with 0.5 and 5 molar equivalents of Cu^{2+} added. Shown are the raw ESEEM traces (black) with fitted exponential background decay (red, left), the background-corrected traces (middle), and the corresponding absolute (magnitude) spectra after FFT (right).

HYSCORE with 0.5 molar equivalents of Cu^{II}

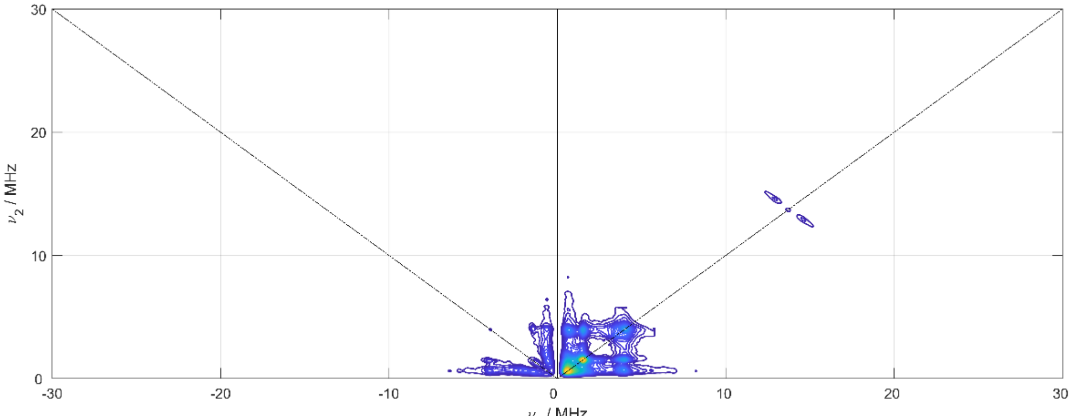

HYSCORE with 5 molar equivalents of Cu^{II}

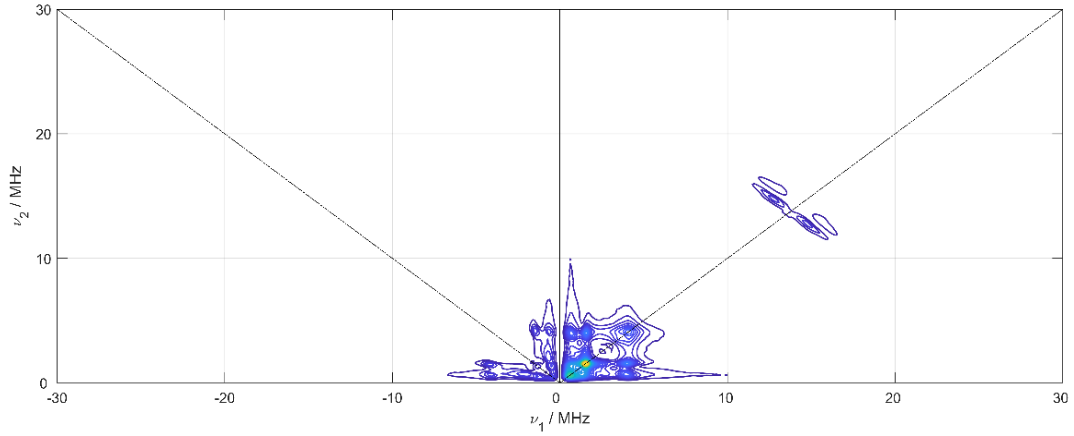

Figure S11. Individual HYSCORE spectra of the pseudo-titration series with 0.5 and 5 molar equivalents of Cu^{2+} added.

Table S5 Distances between the Cu^{2+} bound at different sites, measured using PyMOL. All the distances are provided in Å.

Sites	ATCUN (H3)	Site B (H9)	Site A (H67 / H246)	H247	H317
ATCUN (H3)					
Site B (H9)	11.6				
Site A (H67 / H246)	20.1	17.4			
H247	35.2	24.8	23.8		
H317	48.8	43.4	40.9	42.2	
H509	72.4	65.8	55.5	45.7	76
